# Spontaneous and TMS-related EEG changes as new biomarkers to measure anti-epileptic drug effects

**DOI:** 10.1038/s41598-022-05179-x

**Published:** 2022-02-04

**Authors:** Andrea Biondi, L. Rocchi, V. Santoro, P. G. Rossini, G. N. Beatch, M. P. Richardson, I. Premoli

**Affiliations:** 1 Division of Neuroscience, Department of Basic and Clinical Neuroscience, Institute of Psychiatry, Psychology and Neuroscience, King’s College London, Maurice Wohl Clinical Neuroscience Institute, Ground Floor (G.33.08), 5 Cutcombe Road, Camberwell, London, SE5 9RX UK; 2 Department of Clinical and Movement Neurosciences, UCL Queen Square Institute of Neurology, University College London, London, UK; 3 Xenon Pharmaceuticals Inc., Burnaby, Canada; 4 Department of Medical Sciences and Public Health, University of Cagliari, Cagliari, Italy

**Keywords:** Neuroscience, Biomarkers

## Abstract

Robust biomarkers for anti-epileptic drugs (AEDs) activity in the human brain are essential to increase the probability of successful drug development. The frequency analysis of electroencephalographic (EEG) activity, either spontaneous or evoked by transcranial magnetic stimulation (TMS-EEG) can provide cortical readouts for AEDs. However, a systematic evaluation of the effect of AEDs on spontaneous oscillations and TMS-related spectral perturbation (TRSP) has not yet been provided. We studied the effects of Lamotrigine, Levetiracetam, and of a novel potassium channel opener (XEN1101) in two groups of healthy volunteers. Levetiracetam suppressed TRSP theta, alpha and beta power, whereas Lamotrigine decreased delta and theta but increased the alpha power. Finally, XEN1101 decreased TRSP delta, theta, alpha and beta power. Resting-state EEG showed a decrease of theta band power after Lamotrigine intake. Levetiracetam increased theta, beta and gamma power, while XEN1101 produced an increase of delta, theta, beta and gamma power. Spontaneous and TMS-related cortical oscillations represent a powerful tool to characterize the effect of AEDs on in vivo brain activity. Spectral fingerprints of specific AEDs should be further investigated to provide robust and objective biomarkers of biological effect in human clinical trials.

## Introduction

The development of a high percentage of central nervous system (CNS) active drugs could fail due to safety concerns and toxicology issues in preclinical studies. Then, the small number of molecules that proceed through the pipeline into human research can encounter further failure due to lack of efficacy in clinical trials. These challenges to CNS drug development have caused a clear reduction in new therapeutic products in this area. Robust and objective biomarkers which can target engagement in the human brain are a key to increase the probability of successful development of new compounds in human trials^1^. The evaluation of pharmacodynamic properties in vivo can be achieved with positron emission tomography (PET) or drug distribution to the CNS by analysing cerebrospinal fluid (CSF) samples; however, these methods are invasive, expensive and not always available. For all these reasons, the development and validation of simple, fast and reliable markers is a paramount challenge.

Transcranial magnetic stimulation (TMS) is a non-invasive brain stimulation technique which, in combination with electroencephalography (EEG), enables a fast and accurate assessment of human brain excitability in health^2,3^ and pathological conditions^4^. TMS-EEG output measures can be interrogated in the time^5^ and time–frequency domains^6^, providing different information about cortical processes^7^. The EEG responses to TMS averaged in the time domain are called TMS-evoked EEG potentials (TEPs), which are a reliable alternating sequence of positive (P) and negative (N) peaks at approximately 25 (P25), 45 (N45), 60 (P60), 100 (N100) and 180 (P180) milliseconds after stimulation of the human primary motor cortex (M1)^5^*.* Time–frequency decomposition of the TMS-EEG signal reveals TMS-induced oscillations which, in contrast with the TEP, contain information not necessarily phase locked to the stimulus^8^. Their typical profile following M1 stimulation is characterized by an early increase of delta, theta, alpha and beta band power up to 200 ms, followed by alpha and beta suppression^9^ (often termed de-synchronization) with a final increase in beta power^10^.

Changes in TEP amplitude has emerged as an in-vivo method to measure the effects of drugs acting on the human brain. Pharmacological studies investigating the inhibitory GABAergic pathways showed that the N45 and N100 peaks are associated with GABA-A^11,12^ and GABA-B^11^ receptor-mediated neurotransmission, respectively. Peaks at 30, 45 and 180 ms are sensitive to the effects of antiepileptic drugs (AEDs) targeting voltage-gated sodium channels blockers (i.e. Lamotrigine and carbamazepine) and type 2A synaptic vesicles (i.e. Levetiracetam)^13–15^. More recently, TEPs were implemented in a commercial Phase I clinical trial to evaluate cortical excitability impact of XEN1101, a novel AED which potentiates the open state of potassium KCNQ2/3 channels^16^, which suppressed peaks at 30, 45 and 180 ms after TMS pulse. Another study investigated the effect of other AEDs on spontaneous EEG oscillations, showing how Carbamazepine increases beta band power, whereas Tiagabine increases broadband EEG power^15^. Furthermore, the effects of specific GABAergic drugs, such as Alprazolam, Baclofen, Diazepam and Zolpidem on oscillatory responses have been studied with TMS-induced oscillations. Results showed that TMS-induced power changes may involve different GABAergic-inhibitory mechanisms^17^.

Despite growing knowledge about the effects of AEDs on brain oscillations and TEPs, their impact on TMS related brain oscillations has not been systematically explored and more studies are needed to better understand the effects of these medications on brain connectivity and excitability. Here, to provide more evidence and support for the use of these measurements to guide drug development, we tested how three different AEDs affect TMS-related brain oscillatory activity. In parallel, we have also explored their modulation of spontaneous brain oscillations. Data have been acquired in two previous TMS-EEG studies, where the effects of Lamotrigine, Levetiracetam (experiment 1)^13^ and XEN1101 (experiment 2)^14^ were shown on TEPs only. Results show that different AEDs induce specific changes in brain oscillatory activity measured by resting EEG and TMS-EEG. Therefore, together with TEPs, spontaneous and TMS-related oscillations are of great potential value for inferring mechanisms and confirming specific target engagement.

## Methods

### Subjects

Fifteen (mean age 25.2 ± SD 4.62) and twenty (mean age 26.6 ± 5.9) healthy male volunteers were recruited for experiments 1 and 2, respectively. Subjects were all right-handed, according to the Edinburgh Handedness Inventory (laterality score ≥ 75%)^18^. Exclusion criteria included intake of CNS active medications, recent use of any kind of drugs (including nicotine and alcohol), neurological and psychiatric disorders, and contraindications to TMS or to medications used in the study (Levetiracetam/Lamotrigine/XEN1101). Experiment 1 was approved by King’s College London Research Ethics Committee Research (CREC), which was performed in accordance with relevant guidelines and regulations. Experiment 2 XEN1101 clinical trial [ClinicalTrials.gov Identifier: NCT03468725, registration date 02/03/2018)] was approved by the Medicines and Healthcare products Regulatory Agency (MHRA) in London. All participants signed a written informed consent before undergoing experimental procedures.

### Experimental design and procedure

The experimental protocols, TEP and RMT drug-induced modulations have already been published in our previous reports^13,14^. In experiment 1, a single oral dose of Lamotrigine (300 mg), a voltage-sensitive sodium channel blocker^19^, or Levetiracetam (3000 mg), a specific binder of synaptic vesicle protein 2A (SV2A)^20^, or placebo, were administered on separate occasions a week apart. In experiment 2, the novel selective positive allosteric modulator of potassium channel KCNQ2/3 (Kv7.2/7.3), XEN1101 (20 mg), in development for treatment of focal epilepsy by Xenon Pharmaceuticals Inc.^16^, or placebo, were administered on separate occasions a week apart. Both studies followed a pseudo-randomized, double blinded, crossover design.

A TMS-compatible EEG system (BrainAmp MRplus; Brain Products) and Magstim 200^2^ (Magstim Company Limited, Whitland, UK) TMS stimulator with a monophasic current waveform connected to a 90 mm figure-of-eight coil were used in both experiments. The EEG signal was acquired with a 64-electrode EasyCap for experiment 1 (EasyCap 64Ch; Brain Products) and a 64-electrode Multitrodes Cap (Multitrodes, BrainCap-Fast'n Easy) for experiment 2. Electrodes were arranged accordind to the International 10–20 montage with channel AFz as ground and FCz as reference, hardware-filtered between DC and 1000 Hz and digitized with a sampling rate of 5 kHz. An impedance below 10 kΩ was kept throughout the experiments during the recording.

MEPs were recorded through surface EMG electrodes (Ag–AgCl cup electrodes) in a belly-tendon montage. The EMG signal was recorded using the Signal software (Cambridge Electronic Design). The EMG raw signal was amplified (Digitimer D360 8-channel amplifier), bandpass filtered (20 Hz—2 kHz) and digitized at an A/D rate of 10 kHz (CED Micro 1401; Cambridge Electronic Design). The coil was oriented with the handle pointing backwards and 45° away from the midline, to induce current in the brain oriented from lateral-posterior to anterior-medial^21^. At the beginning of each stimulation session, after identification of the first dorsal interosseous muscle (FDI) hotspot in the left M1, the resting motor threshold (RMT) was measured as the minimum intensity able to elicit a 50 μV peak-to-peak motor evoked potential in 5 out of 10 consecutive trials^22^. Using a 100% RMT intensity, 150 single pulses were delivered over the M1 hotspot, with a 4 s interstimulus interval and a variance of 20% to avoid adaptation. A masking noise was used to avoid possible contamination of the EEG signal by auditory potentials induced by the TMS click^23,24^. The same TMS intensity was used for baseline and post-drug stimulation, for two main reasons. The first reason was that, by adjusting the stimulation intensity to a new RMT value, possible power changes ascribable to the effects of AEDs would have been confounded by effects of the different stimulation intensity. The second was to keep the artefact induced by scalp muscle activation by TMS constant, since it is known that the latter can partly contaminate TMS-EEG responses. However, the effects of AEDs on RMT were separately investigated by re-measuring RMT after drug intake^13,14^.

**Figure 1:**
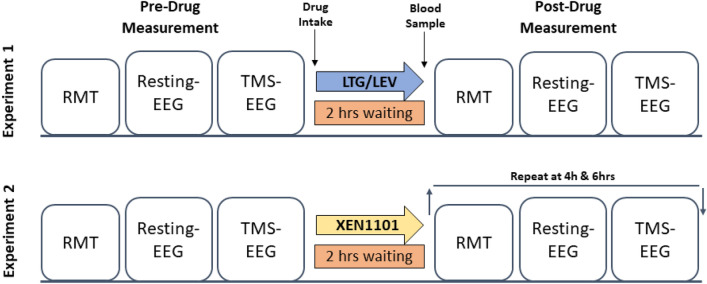
Experimental protocol and timeline. Lamotrigine, Levetiracetam and placebo were administered on separate occasions in experiment 1 and XEN1101 and placebo on separate occasions in experiment 2. Both studies followed a randomized and crossover design. RMT, Resting-state EEG and TMS-EEG sessions were recorded for each subject at baseline (pre-drug measurement) and at 2 (experiment 1 and 2), 4 and 6 h (experiment 2) after drug intake. Five minutes before each post-drug measurement a blood sample was taken to measure drug plasma concentration.

TMS-EEG and resting EEG recordings were performed at baseline (pre-drug) and at 2 (experiment 1 and 2), 4 and 6 h (experiment 2) after drug intake (Fig. 1). Five minutes before each post-drug measurement a blood sample was taken for every subject to evaluate drug plasma concentration. XEN1101 showed a pharmacokinetic profile characterized by a prolonged absorption and XEN1101 was detectable (< 8.22 ng/mL) a week after administration, during the placebo experiment in those subjects who had placebo at the second visit^14^. Therefore, to investigate XEN1101 effects, we selected post-dose measures for TMS-EEG and resting EEG measurements taken during the highest drug exposure (> 8.22 ng/mL) at the time of the post-drug TMS session (Supplementary Table 1). The same timing of post-drug measurement was chosen to perform a time-matched placebo comparison at individual level.

### Analysis of TMS-related spectral perturbation (TRSP)

To investigate the effects of different AEDs on TMS-related spectral perturbation (TRSP), the whole EEG signal was analysed using MATLAB® (Mathworks Ltd, USA, R2012b) (The Mathworks Inc.) and FieldTrip toolbox^25^. TRSP is defined as the event-related changes in spectral power over time in a broad frequency range and it takes into account the phase-locked and non-phase locked EEG perturbations induced by TMS^8,26^.

After excluding trials with prominent eye movements, blinks, and muscle artefacts (on the basis of visual inspection), data were segmented into epochs of 1 s length before and after the TMS pulse, and linearly interpolated for ± 10 ms to remove the TMS artefact. Bad channels were removed from the EEG, and the signal was reconstructed by interpolating the surrounding electrode signals. Data were then notch-filtered (50 Hz) and down-sampled to 1 kHz. Independent Component analysis (ICA) was applied to remove TMS-related artifacts (i.e., cranial muscle response, recharge of capacitors, and related exponential decay artifacts^27–29^), as well as further muscle and ocular activity. Finally, remaining data were re-referenced to the linked mastoids, baseline corrected (from − 1000 to − 50 ms) and band-pass filtered (1–80 Hz).

The time–frequency representations were calculated by applying a Hanning taper windowed fast Fourier transform (FFT) with frequency-dependent window length (width: 3.5 cycles per time window, time steps: 10 ms, frequency steps: 1 Hz from 2 to 45 Hz)^26^. We then applied a single-trial z-transformation, based on the mean and standard deviation of the full-length trial and baseline-corrected by subtracting the mean value of the baseline period (from – 1000 to 50 ms before TMS)^17^. For each drug condition in both experiments, TMS-related oscillations were classified in delta (2–4 Hz), theta (4–7 Hz), alpha (8–12 Hz), beta (13–30 Hz) and gamma (30–45 Hz) frequency bands.

### Analysis of resting-state EEG

To investigate the effects of the AEDs on spontaneous brain oscillations, 3 min of eyes closed resting state EEG data were analyzed. Data were segmented into 2 s epochs, band-pass filtered (2–80 Hz), down-sampled (1 kHz) and an automatic artefact rejection as implemented in Fieldtrip was conducted to remove epochs containing eye movements or muscle/movement artifacts. Data were visually inspected, and epochs contaminated by residual artefacts were removed manually. The cleaned resting-state EEG data were then re-referenced to the average of all EEG channels. Power spectra were determined via FFT for frequency bins from 2 to 45 Hz in steps of 0.5 Hz, and spectra were averaged across segments and EEG channels. As for TMS-related oscillation, resting-state EEG power was classified for discrete frequency bands, i.e., delta (2–4 Hz), theta (4–7 Hz), alpha (8–12 Hz), beta (13–30 Hz) and gamma (30–45 Hz) frequency bands.

## Statistics

To investigate the effects of different AEDs on the power of TMS-related oscillations and resting state EEG, multiple dependent sample t tests at the individual electrode level within each drug condition (Lamotrigine, Levetiracetam and placebo in experiment 1; XEN1101 and placebo in experiment 2) were applied. Specifically, paired *t *tests were applied to compare (1) post- versus pre-drug data within the same drug condition and (2) between post-drug (or placebo) conditions, for each electrode in a selected a region of interest (ROI) that was composed of 27 channels around the stimulation site and the corresponding contralateral site (FC1, FC3, FC5, C1, C3, C5, CP1, CP3, CP5, P1, P3, P5, Cz, CPz, Pz, FC2, FC4, FC6, C2, C4, C6, CP2, CP4, CP6, P2, P4, P6) and for each frequency of interests: delta (2–4), theta (4–7 Hz), alpha (8–12 Hz) beta (13–30 Hz) and gamma (30–45 Hz). TMS-related oscillations were compared in a single time of interest from 30 to 800 ms. Spectral fingerprints were corrected for multiple comparisons (i.e. electrodes, time and frequency points) and a cluster-based permutation analysis was applied^30^. T-values exceeding an a priori threshold of p = 0.05 were clustered based on adjacent time bins and neighboring electrodes. Cluster-level statistics was calculated by taking the sum of the t values within every cluster and each statistical comparison was done with respect to the maximum values of summed t-values. Cluster-based permutation tests were performed as described above to check differences between pre-drug states within the same experiment, both for TMS-EEG and resting EEG signals, separately.

## Results

For experiment 1 fifteen male subjects aged 19–34 years (mean age ± standard deviation [SD] 25.2 ± 4.62 years) were enrolled. Only one participant was unable to complete the TMS-EEG procedure after the intake of Lamotrigine due to side effects (i.e. vomiting), leaving a total number of 14 subjects^13^. Twenty subjects (mean age ± standard deviation (SD) of 26.6 ± 5.9 years (range 19–40 years) took part in experiment 2. XEN1101 showed a prolonged absorption and long elimination half-life, hence 4 participants did not reach XEN1101 concentrations higher than the carry-over observed in the placebo arm (8.2 ng/mL) at the time of TMS testing^14^.

### Antiepileptic drugs effects on TMS-related oscillations

At baseline, prior to ingestion of drug or placebo, single-pulse TMS induced a specific pattern consisting of an early increase in power in the theta, alpha and beta bands; the latter showed a later decrease and a final increase in power, as described previously^9,10,17^. The baseline comparison of experiment 2 showed a higher TMS-related increase in beta power from 560 to 680 ms in the pre-placebo compared to pre-XEN1101 (p = 0.04). Finally, placebo did not produce significant changes in the TMS-related spectral profile in any frequency band and for both experiments (all p values > 0.05) (Supplementary Figs. 6, 7). The effects for each drug condition are reported below. Then, we applied the Spearman correlation coefficient to investigate possible correlations between drug-related modulation (Diff = Post minus Pre-drug) of TRSP and the corresponding blood plasma concentration. All the analysis performed did not show any significant correlation (p > 0.05) for each drug condition.

#### Lamotrigine

**Figure 2:**
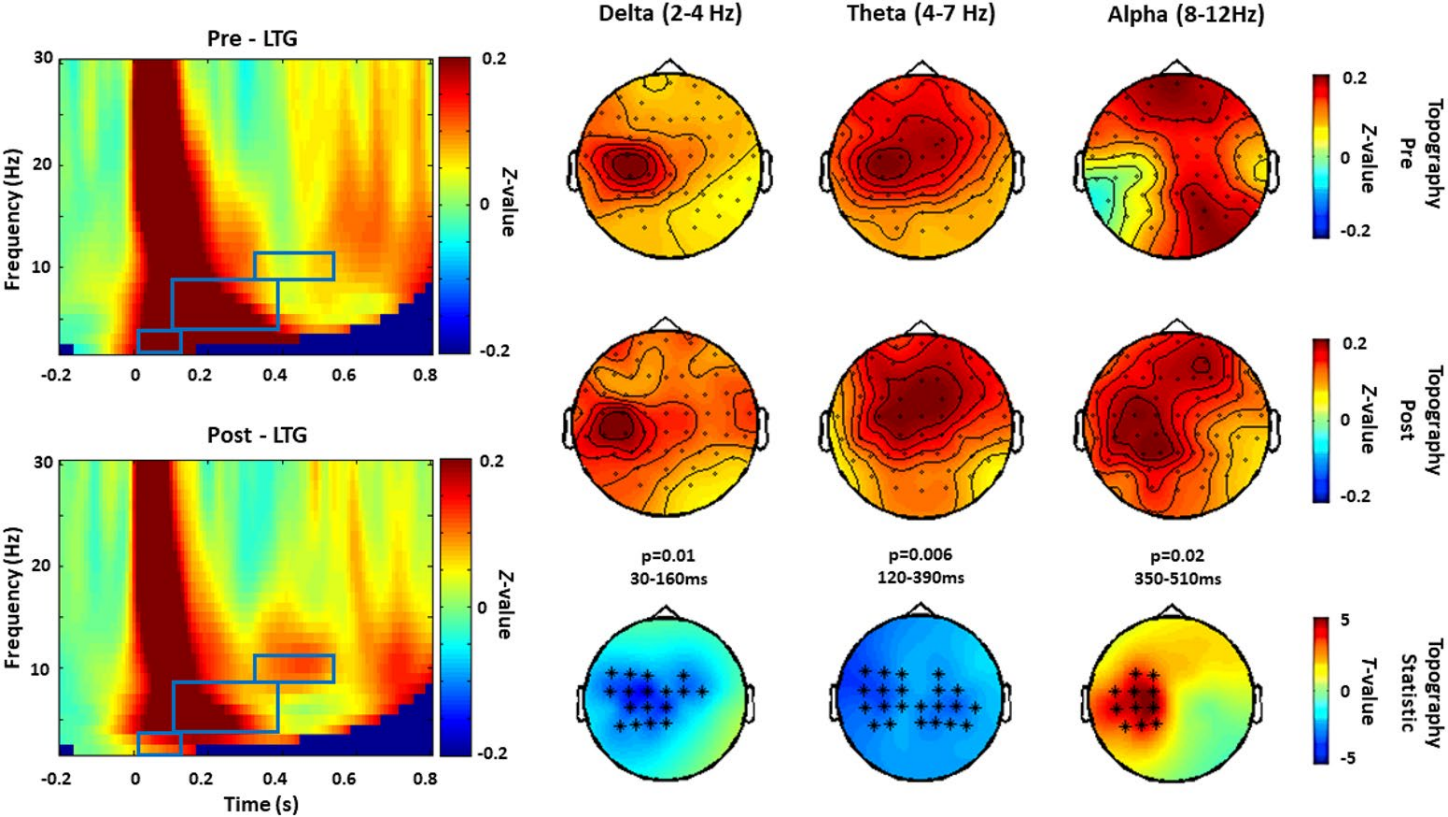
TRSP modulated by Lamotrigine (experiment 1). Grand averages of the time–frequency representation (TFR averaged over ROI channels) of TRSP recorded before (pre) and after (post) the intake of Lamotrigine are shown on the left panel. The blue boxes correspond to the time window when comparison between pre and post conditions showed significant drug effects. Topographical distributions of drug-related effects on delta (p = 0.01, 30–160 ms), theta (p = 0.006, 120–390 ms) and alpha band power (p = 0.02, 350–510 ms) are reported for pre and post drug conditions on the right panel. Significant electrodes within the bilateral 27 electrode ROIs are represented with asterisks in the t-statistic map.

Compared to baseline, Lamotrigine showed a significant decrease in TRSP delta (p = 0.01, 30–160 ms), theta (p = 0.006, 120–390 ms) and a significant increase of the alpha band power (p = 0.02, 350–510 ms). The effect on delta and alpha bands appears to be located over channels close to the stimulated area (left M1), while theta power reduction occurred in a larger area, including bilateral central and parietal electrodes (Fig. 2). Comparisons between post-Lamotrigine and post-placebo conditions supported the significant decrease in delta (p = 0.003, 30–160 ms) and theta (p = 0.04, 120–220) power and the increase in alpha power (p = 0.03, 390–510).

**Figure 3:**
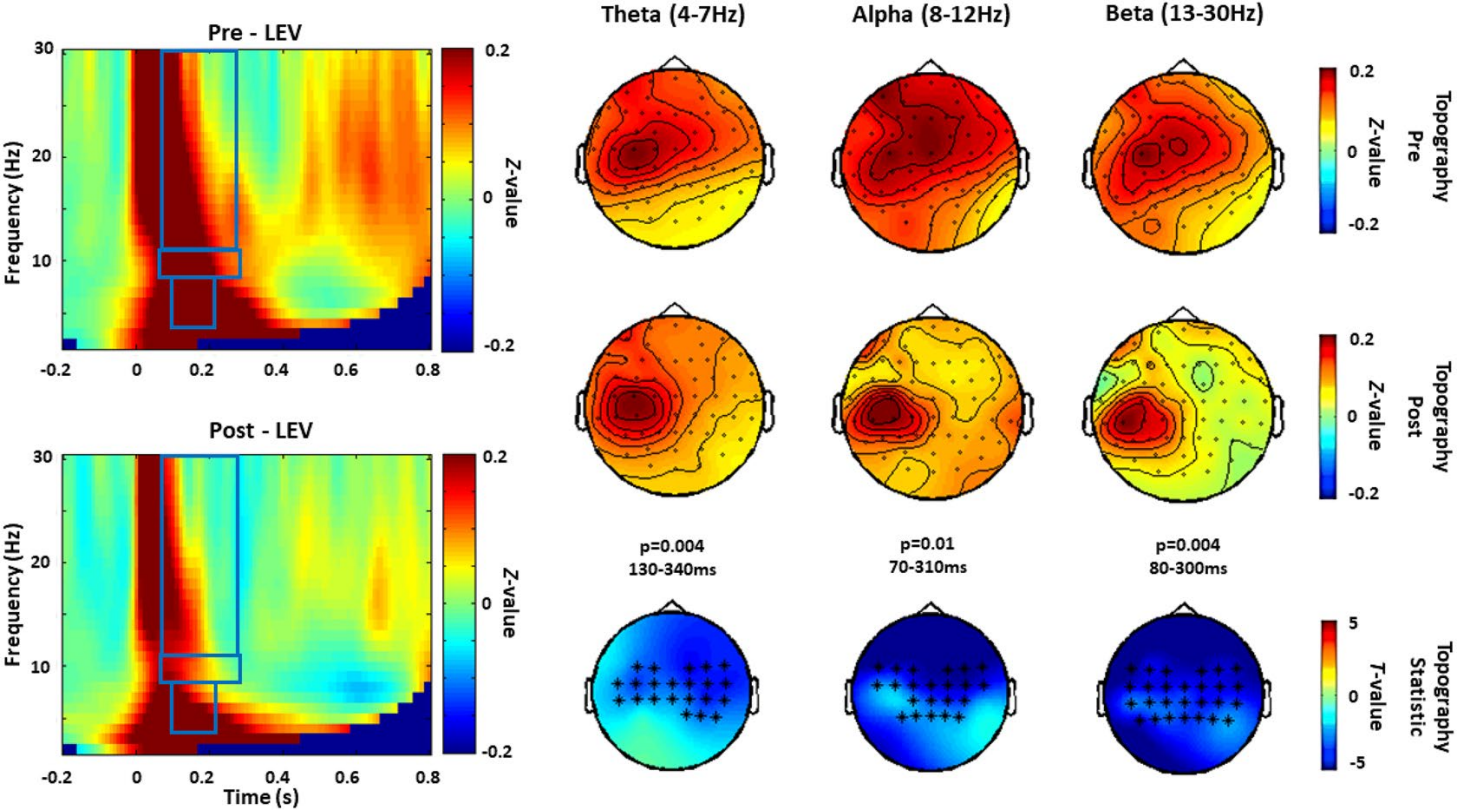
TRSP modulated by Levetiracetam (experiment 1). Grand averages of the time–frequency representation (TFR averaged over ROI channels) of TMS-related oscillations recorded before (pre) and after (post) the intake of Levetiracetam are shown on the left panel. The blue boxes correspond to the time and frequency windows when comparisons between pre and post conditions showed significant drug effects. Topographical distribution of drug-related effects on theta (p = 0.004, 130–340 ms) and alpha (p = 0.01, 70–310 ms) and the beta (p = 0.004, 80–300 ms) band power are reported for pre and post drug conditions on the right panel. Significant electrodes within the bilateral 27 electrode ROIs are represented with asterisks in the t statistic maps.

**Figure 4:**
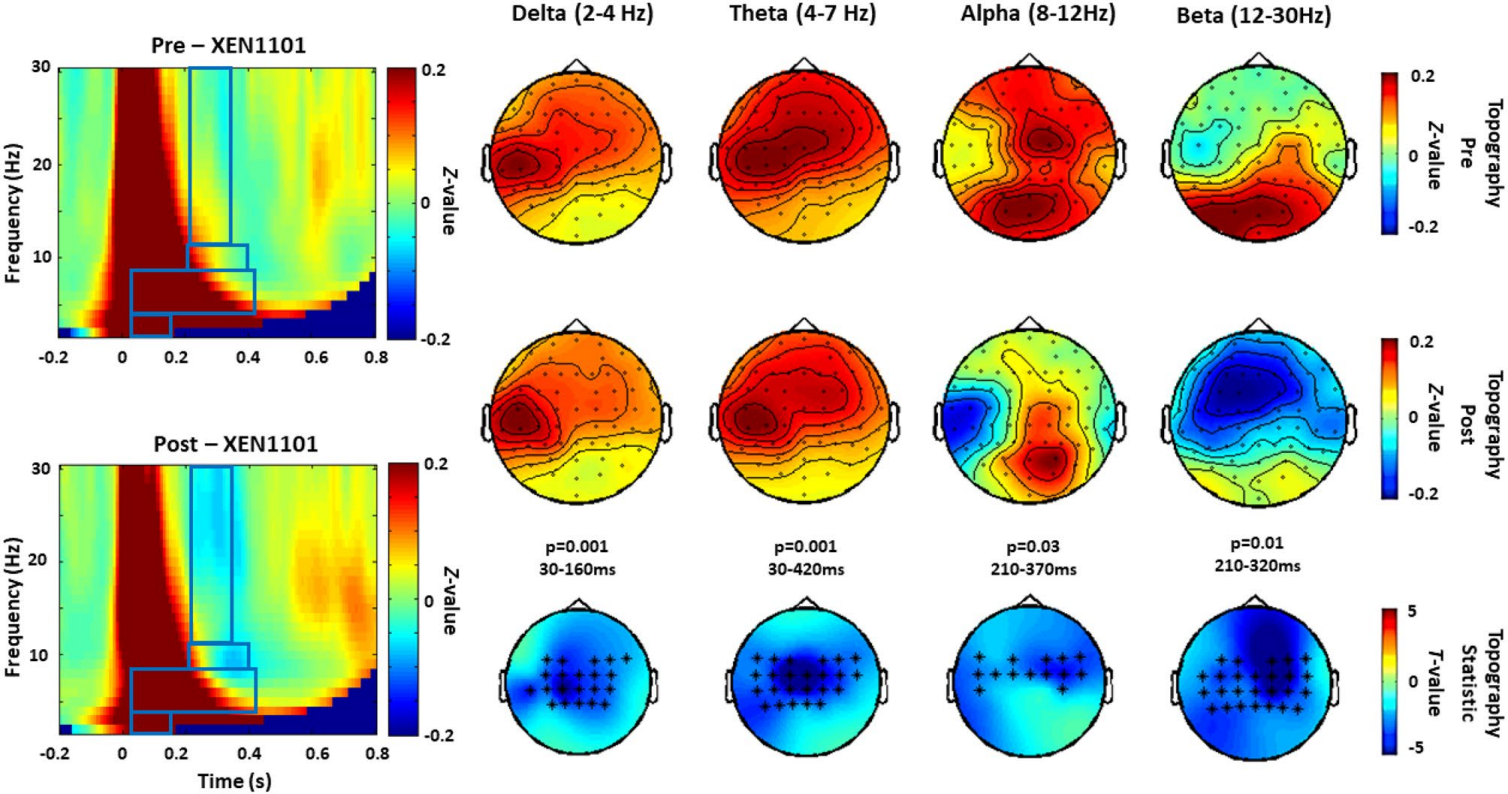
TRSP modulated by XEN1101 (experiment 2). Grand averages of the time–frequency representation (TFR averaged over ROI channels) of TRSP oscillations recorded before (pre) and after (post) the intake of XEN1101 are shown on the left panel. The blue boxes correspond to the time and frequency windows when comparisons between pre and post conditions showed significant drug effects. Topographical distribution of drug-related effects on the of delta (p = 0.001, 30–160 ms), theta (p = 0.001, 30–420 ms), alpha (p = 0.03, 210–370 ms) and beta (p = 0.01, 210–320 ms) band power are reported for pre and post drug conditions on the right panel. Significant electrodes within the bilateral 27 electrode ROIs are represented with asterisks in the t-statistic maps.

#### Levetiracetam

As shown in Fig. 3, Levetiracetam induced a reduction in theta (p = 0.004, 130–340 ms), alpha (p = 0.01, 70–310 ms) and beta (p = 0.004, 80–300 ms) power. This occurred in a large area, including bilateral central and parietal electrodes.The comparison between post-Levetiracetam and post-placebo confirmed the suppression of theta (p = 0.009, 70–320 ms), alpha (p = 0.007, 30–360 and p = 0.02, 380–780) and beta power (p = 0.03, 50–180).

#### XEN1101

XEN1101 induced a significant suppression of delta (p = 0.001, 30–160 ms), theta (p = 0.001, 30–420 ms), alpha (p = 0.03, 210–370 ms) and beta power in a large area including bilateral central and parietal electrodes (p = 0.01, 210–320 ms) (Fig. 4). All the effects observed were supported by comparisons against post-placebo conditions: suppression of delta (p = 0.001, 30–160), theta (p = 0.01, 30–250), alpha (p = 0.04, 250–350) and beta (p = 0.02, 210–250) were all significant (p < 0.05).

### Antiepileptic drugs effects on resting state EEG

Before drug intake, in both experiments, the cluster-based permutation comparisons between pre-drug or pre-placebo conditions did not show significant differences in the resting oscillatory power in all frequency bands (all p > 0.05). Finally, placebo did not produce significant changes on resting-state EEG spectral profile in any frequency band and for both experiments (all p > 0.05) (Supplementary Figs. 7, 8). Below we report effects for each drug condition.

#### Lamotrigine

**Figure 5:**
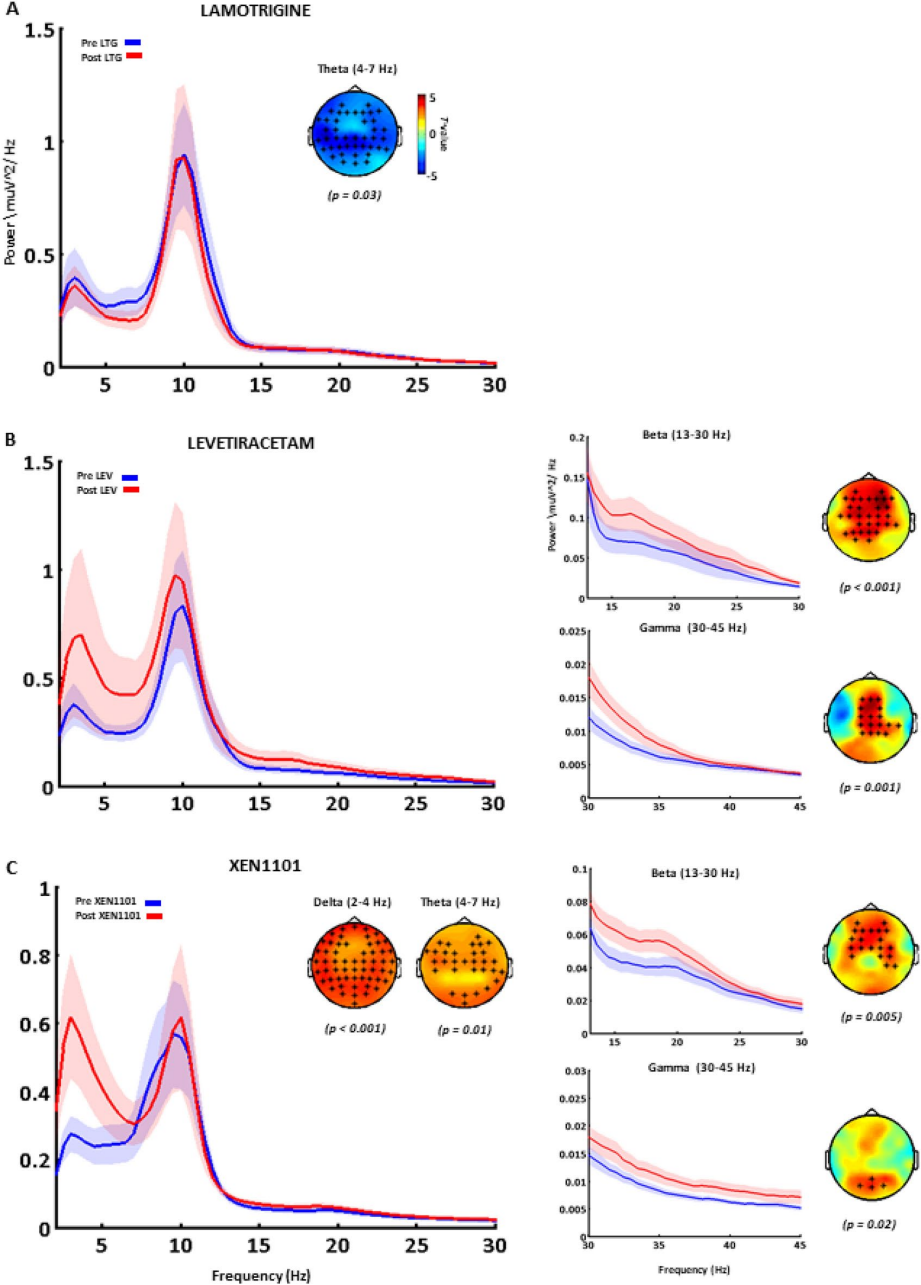
The effects of antiepileptic drugs on resting-state EEG oscillations. Grand-averaged power spectrums calculated on the average of all channels are reported before (pre, blue) and after (post, red) the intake of Lamotrigine (**a**), Levetiracetam (**b**) and XEN1101 (**c**). For each drug condition, significant differences are indicated with the respective topographical distribution of t-values where significant channels are indicated with asterisks. Lamotrigine (**a**) decreases theta power (p = 0.03); Levetiracetam (**b**) increases theta (p = 0.03), beta (p < 0.001) and gamma (p = 0.001) power; XEN1101 increases delta (p < 0.001), theta (p = 0.01), beta (p = 0.005) and gamma (p = 0.02) power. The significant modulation of beta and gamma power are shown for each drug in a zoomed power spectrum (panels on the right; averaged over significant channels for Levetiracetam and XEN1101, respectively).

Compared to pre-drug intake, Lamotrigine significantly decreased spontaneous theta band power (p = 0.03) over all sensors compared and had no effect on other frequency bands (p > 0.05; Fig. 5A). The same significant modulation was confirmed when comparing post-Lamotrigine versus post-placebo (p = 0.01).

#### Levetiracetam

Compared to baseline, a single dose of Levetiracetam significantly enhanced beta (p < 0.001) and gamma power (p = 0.001) over medial frontal and parietal electrodes and theta band power at a right lateral cluster (p = 0.03; Fig. 5B). The modulatory effect of Levetiracetam on beta and gamma band oscillations was supported by the comparison with the post-placebo condition (p = 0.03 and p = 0.04); however, differences on theta and alpha bands were not significant (p > 0.05).

#### XEN1101

In subjects showing good XEN1101 exposure (plasma levels > 8.22 ng/mL) at the time of assessments, resting state oscillatory activity was significantly modulated. Specifically, a significant increase in delta frequency power (p < 0.001) in frontal, parietal and occipital electrodes, a significant power increase of theta and beta bands in medial frontal and parietal electrodes (p = 0.01 and p = 0.005) and an increase in gamma power (p = 0.02) in the occipital electrodes were found (Fig. 5C). All these effects were confirmed when XEN1101 was compared with the post-placebo condition. The analysis showed an enhanced power of delta (p < 0.001), theta (p = 0.04), beta (p < 0.01) and gamma (p = 0.02) frequency bands.

## Discussion

Our results extend previous studies where we demonstrated that non-invasive electrodiagnostic techniques, such as TMS-EEG, can inform our understanding of mechanisms of action of drugs acting on the CNS. Here we investigated the modulation of TMS-related cortical oscillations and resting-state EEG power by AEDs with different mechanisms of action in healthy volunteers.

In the TMS-related oscillation analysis, delta power was decreased by Lamotrigine and XEN1101, theta was suppressed by all AEDs, whereas alpha power was suppressed by Levetiracetam and XEN1101, while it was increased only by Lamotrigine. Finally, beta power was reduced by Levetiracetam (80–300 ms) and by XEN1101 from 210 to 320 ms, where typically beta is suppressed (or de-synchronized) by TMS^17^. Signatures at the resting state EEG level showed a common pattern for Levetiracetam and XEN1101, with an increase in theta, beta and gamma power, in contrast with suppression of theta power by Lamotrigine. Finally, delta power was increased by XEN1101 only.

### Cortical oscillations to study neural processes

Cortical oscillations have been widely studied in the EEG literature and specific frequency bands have been associated with distinct behaviours or cognitive functions^31,32^; their pattern may also reflect dysfunction in neural networks in a pathological brain^33^. Cortical oscillations can be observed at rest, when no task is performed, or they can be induced by a given stimulus (i.e., TMS).

### TMS-related power oscillations to investigate the effects of antiepileptic drugs

Changes in cortical oscillations following TMS on M1 involve an increase in power of delta, theta, alpha and beta frequency bands in a period up to 300 ms after TMS, followed by a beta reduction (de-synchronization) and a subsequent beta increase^10^; despite their reproducibility, the mechanisms underlying this phenomena are still not fully understood. Pharmacological investigations suggested that early power increase (up to 200 ms) and late decrease (200–400 ms) of induced oscillations may be mediated by separate inhibitory mechanisms. As such, early increase in alpha-band power was enhanced by a GABAAR-mediated drive (zolpidem, diazepam and alprazolam) and reduced by GABABR-mediated activity (baclofen), whereas both GABAAR- and GABABR-activity enhanced the reduction of beta-band power. Finally, the GABABR agonist Baclofen enhanced the reduction of alpha-band power^17^.

In the light of the existing literature, we may speculate that the increase of TMS-related alpha oscillation by Lamotrigine is in line with the effects of Diazepam and Zolpidem^17^ and this may suggest an effect mediated by local cortical circuits in which GABA-A synapses have a predominant effect. Lamotrigine, which is also used as a mood stabilizer, does not act directly on GABAAR; however, it was reported to increase the amplitude and the frequency of spontaneous GABAAR-mediated inhibitory postsynaptic currents by increasing GABA release in vitro^34^. Lamotrigine also decreased delta and theta TRSP, showing how this AEDs can also decrease pathological thalamo-cortical synchronization. Previous studies in patients with epilepsy showed how LTG can caused a significant diffuse increase in the faster frequencies and decrease in the slower activities^35,36^. Finally, in TMS-EEG experiments, Lamotrigine increased the N45 TEP component^13^, which is related to GABAergic neurotransmission^11^.

Levetiracetam and XEN1101 showed a similar pattern of suppression of TMS-related theta activity up to ~ 300 ms. During the same time window, Levetiracetam extended the suppression also over alpha and beta while XEN1101 reduced beta power at 210–300 ms, similar to Baclofen, Diazepam and Alprazolam, and also alpha at 210–370, like Baclofen. The exact mechanisms driving the TMS-related de-synchronization/rebound of beta oscillations over sensorimotor cortices is not well understood; however, it may be a direct consequence of the transcranial activation of beta oscillation generating cortico-cortical and cortico-subcortical circuits. The hypothesis that the motor thalamus facilitates cortically-generated TMS-related beta oscillations through cortico-subcortico-cortical feedback loops is supported by a study conducted on a patient with Parkinson’s disease who had undergone unilateral surgical lesioning of the ventrolateral nucleus of the thalamus^37^. Beta oscillations obtained in response to pulses applied over the intact hemisphere was higher than that obtained in healthy controls. The authors proposed that thalamotomy served to reduce the abnormally high TMS-related beta oscillations^37^. Another study demonstrated that patients with severe disorders of consciousness failed to show TMS related alpha and beta desynchronization^38^. This pattern may reflect a consequence of the breakdown of cortico-cortical neuronal connectivity. Interestingly, TMS-evoked oscillations at the motor area at longer time intervals (400–700 ms) were abnormally increased in patients with schizophrenia^39^, suggesting a possible disinhibition of the motor cortex.

It is important to highlight that TMS-evoked EEG potentials were modulated in a similar way by Lamotrigine and Levetiracetam. To further explore this finding, it is useful to refer to a recently developed computational approach which enables the analysis of high-dimensional datasets, to reveal low-dimensional descriptions of effects^40^. A relatively simple model (PARAFAC) was validated to show the joint effect of Levetiracetam and Lamotrigine over channels, time, frequency, subjects and in comparison with pre-drug intake. The model revealed that both drugs suppress oscillations in the alpha range in the occipital region and that this effect was stronger with Levetiracetam. These results demonstrate that time–frequency decomposition may reveal additional relevant features of AEDs effects.

### Spontaneous cortical oscillations to investigate the effects of antiepileptic drugs

Several studies have attempted to address candidate mechanisms of resting-state oscillation generation; for example, some pyramidal neurons in the visual cortex have been shown to engage in spontaneous rhythmic firing due to their intrinsic membrane properties^41^. Oscillations can also occur in loops involving the thalamus, other cortical areas, subcortical structures, or the spinal cord. Finally, a number of studies have shown that an isolated cortical network can produce stable oscillatory discharges and that inhibitory interneurons play the critical orchestrator role by periodically silencing bursts from excitatory cells^42,43^. Given that benzodiazepines act on GABA-A receptors to increase inhibitory post-synaptic potentials (IPSPs), they have been used to explore the underlying mechanism and functional role of cortical oscillations. It is well known in clinical practice that, at rest, benzodiazepines enhance beta band power in EEG recorded from the frontal cortex^44^, whereas they often reduce α-band power (usually reported for parieto-occipital regions)^45,46^. Baclofen, a GABA-B receptor agonist which mediates inhibition by increasing K^+^ currents, induces an increase in spontaneous alpha and beta power^17^. Theta oscillations were first discovered in the rabbit hippocampus in 1938^47^. The theta-memory link was later specifically strengthened by studies showing that the phase of theta oscillations modulates synaptic plasticity^48^. Finally, gamma oscillations have been extensively investigated given their influence of cognition and abnormal behaviour in schizophrenia^49^.

Resting state EEG recording has been applied together with AEDs in a series of studies to describe quantitative changes of EEG signals. The effects of Lamotrigine and Levetiracetam have been tested in healthy participants and results showed a decrease of theta and alpha spectral power for Lamotrigine^50^ and no significant modulation for Levetiracetam^51^. The discrepancy with our results for Levetiracetam may result from the EEG acquisition system which used a lower number of electrodes than in our experiment here. However, in patients with epilepsy, Levetiracetam showed a consistent increase of relative beta power^52,53^. Changes in theta and beta bands have been correlated to improved performance in cognitive tests for attention, working memory and executive functions^53^. In patients with Alzheimer’s disease, Levetiracetam produces a reduction in power of low frequency bands (delta) and an increase of beta bands^54^. Similar effects have been observed for the first time with XEN1101. The increase of beta waves particularly in the frontal areas has already been reported in the literature after benzodiazepines intake^44^.

Levetiracetam and XEN1101 increased gamma frequency power over centro-frontal sites, whereas XEN1101 on occipital channels. High-frequency power increase is likely generated through networks in a cycle of GABA-A receptors-mediated alternating inhibition and excitation^55^. Excitatory NMDA receptors contribute to the generation of network oscillations via modulation of both interneuron to interneuron and interneuron to pyramidal neuron transmission. These oscillations are controlled by a specific class of inhibitory interneurons that can be identified based on either their fast-spiking electrophysiological properties or their expression of calcium-binding protein parvalbumin (PV)^56,57^. Kv7.2 and Kv7.3 channels are expressed in regular and fast-spiking interneurons and retigabine, a K^+^ channel opener with similar properties to XEN1101, showed effects on these neural elements^58^. Therefore, we may speculate that XEN1101 potentiates K^+^ currents over interneurons generating activity in the gamma frequency band.

Levetiracetam has been demonstrated to modulate cognitive functions and this effect has been related to theta oscillations^59^; however, there are no published results on gamma oscillations. The gamma power increase induced by Levetiracetam speaks in favour of a possible impact on cognitive functions, given the association between higher gamma synchrony with stronger neural network engagement through establishing correlations with specific functions^49^.

### Limitations

Some limitations to this study should be mentioned. First, due to a lack of similar studies in the literature, it is difficulty to mechanistically interpret our results and more experiments are therefore needed to better understand this field. Second, TMS was delivered at 100% RMT; this means that MEPs were observed in some trials and, therefore, our TEPs could have been contaminated by indirect brain activation due to feedback activity from muscle twitches. However, in previous analyses, we found no evidence that changes in TEPs amplitude covary with those of MEPs^14^. Additionally, TMS-related oscillations show drug-specific fingerprints regardless of specific increases in RMT, as observed in our previous report^17^.

### Conclusion

In conclusion, we showed that measuring phase-locked and non-phase locked EEG brain oscillations may have a great potential as a biomarker of mechanisms of action in studies assessing the effect of AEDs. We believe that in parallel with the other excitability, connectivity and plasticity biomarkers, the implementation of new biomarkers will allow to accelerate early development of new drugs, by revealing that a new drug acts on and modulates a target mechanism of interest, with possible positive implications for patients’ quality of life, health care providers and pharmaceutical companies. For all these reasons, it is crucial to develop a more thorough and systematic account of changes in brain oscillations induced by AEDs. In addition, these results may provide further insight to pathophysiology of other neurological and psychiatric conditions and to the evaluation of neurophysiological basis of cognition.

## Supplementary Information

Supplementary Information.

## Data Availability

The data of Experiment 1 and Experiment 2 are available upon request.
