# Supplementary material for: Spontaneous and TMS-related EEG changes as new biomarkers to measure anti-epileptic drug effects
Source: Sci Rep. 2022 Feb 4;12:1919. doi: 10.1038/s41598-022-05179-x (PMC8817040; doi:10.1038/s41598-022-05179-x)
Supplement: Supplementary file 1 — Supplementary Information. [file 41598_2022_5179_MOESM1_ESM.pdf]

Supplementary Materials

Figure 6 - TRSP modulated by placebo (Experiment 1)

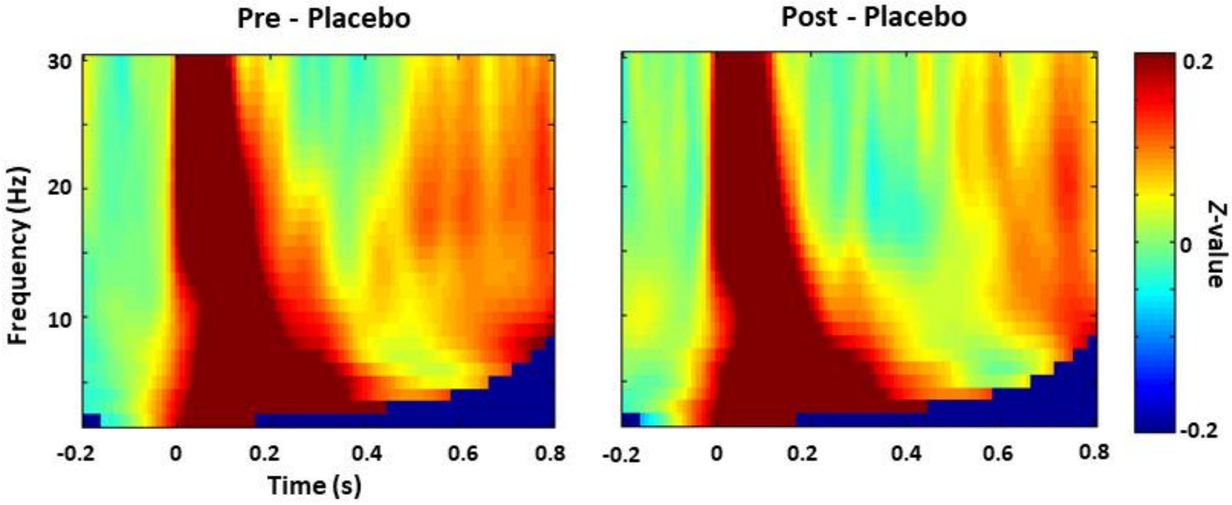

Grand averages of the time-frequency representation (TFR averaged over ROI channels) of TMS-related oscillations pre (left) and post (right) in the placebo condition of experiment 1. Placebo did not produce significant changes in the TMS-related spectral profile in any frequency band and for both experiments (all p values > 0.05).

Figure 7 - TRSP modulated by placebo (Experiment 2)

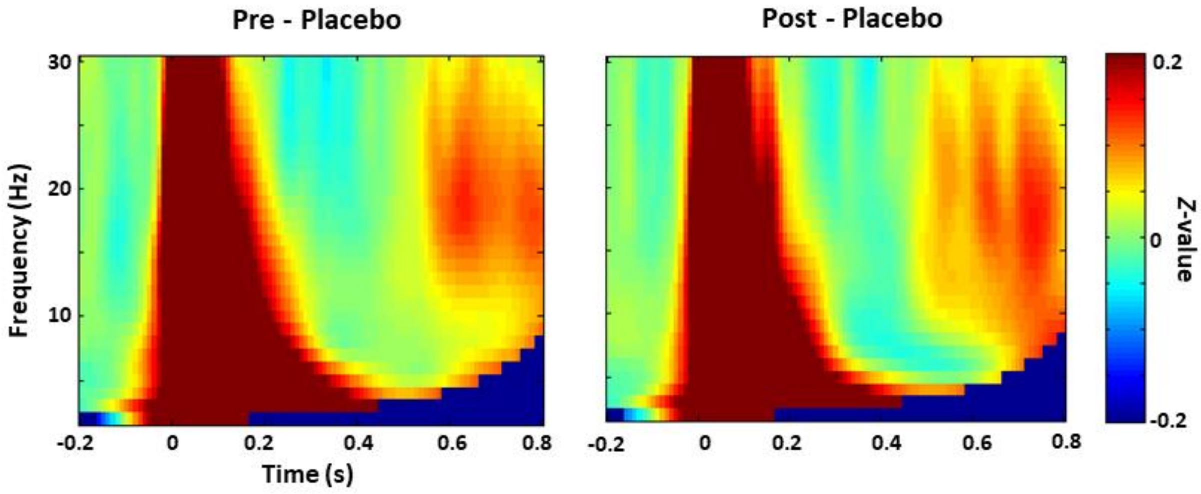

Grand averages of the time-frequency representation (TFR averaged over ROI channels) of TMS-related oscillations pre (left) and post (right) in the placebo condition of experiment 2. Placebo did not produce significant changes in the TMS-related spectral profile in any frequency band and for both experiments (all p values > 0.05).

Figure 8 - The comparison between pre and post placebo resting-state EEG oscillations (Experiment1)

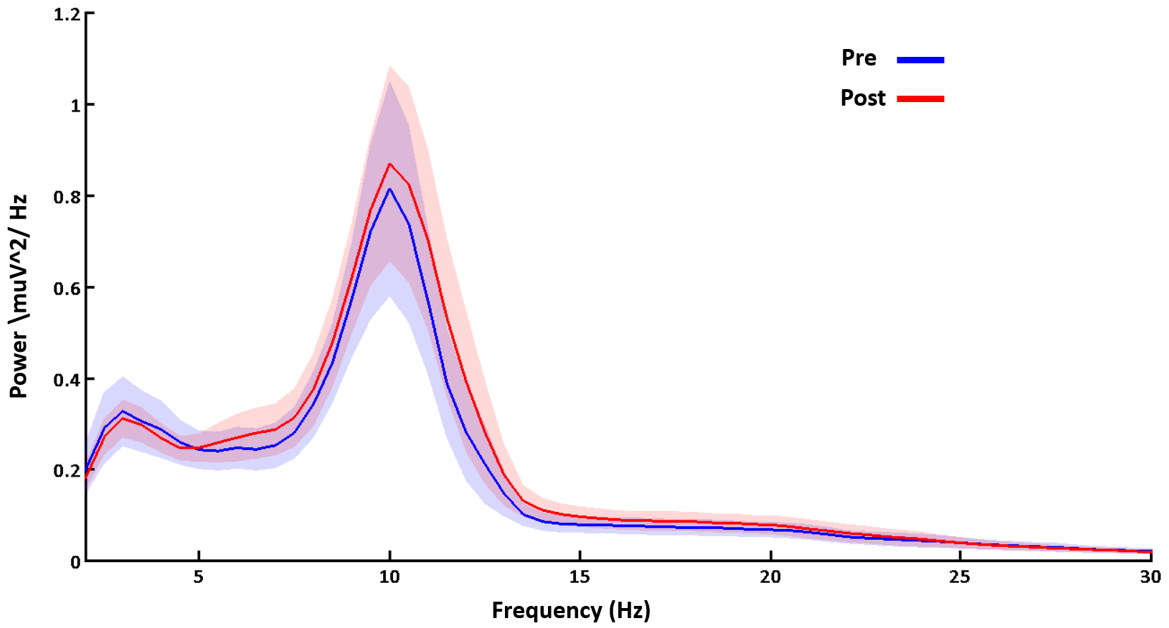

Grand-averaged power spectrums calculated on the average of all channels are reported before (pre, blue) and after (post, red) in the placebo condition of Experiment 1. Placebo did not produce significant changes on resting-state EEG spectral profile in any frequency band and for both experiments (all p>0.05).

Figure 9 - The comparison between pre and post placebo resting-state EEG oscillations (Experiment2)

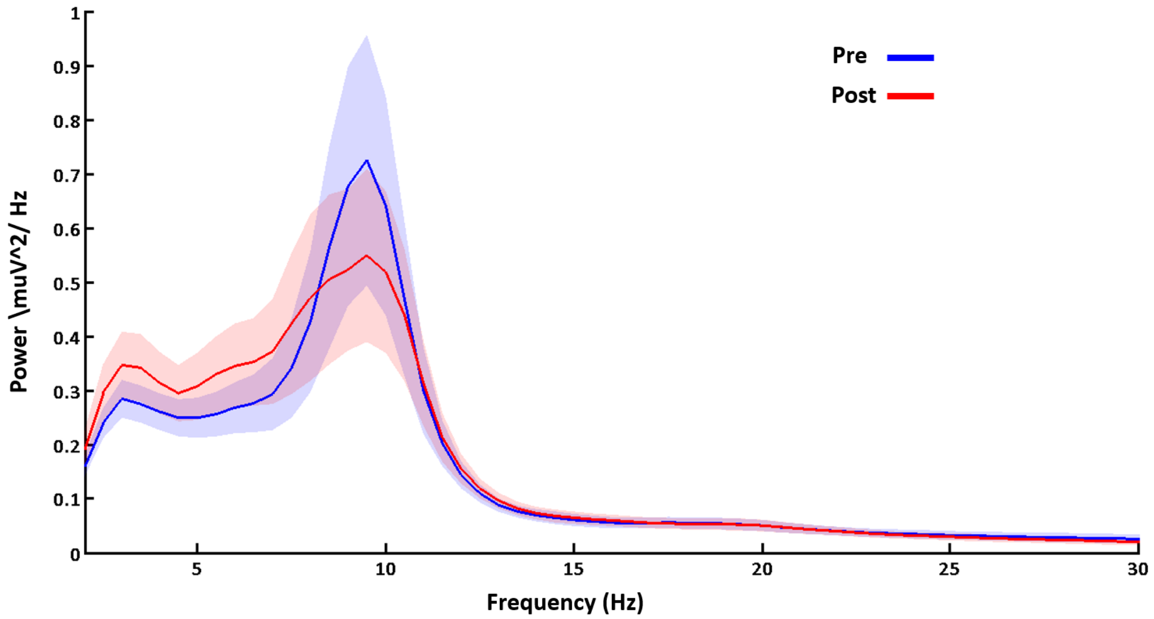

Grand-averaged power spectrums calculated on the average of all channels are reported before (pre, blue) and after (post, red) in the placebo condition of Experiment 2. Placebo did not produce significant changes on resting-state EEG spectral profile in any frequency band and for both experiments (all p>0.05).

Table 1 - XEN1101 Drug Plasma Level Concentration

Subject number	XEN1101 (ng/mL)	Post drug measurement
2	76.5	4 hours
3	47.2	2 hours
4	8.92	4 hours
5	48.4	4 hours
6	16.5	4 hours
7	55.2	2 hours
8	24.5	4 hours
9	47.1	2 hours
12	29.8	4 hours
14	60.7	4 hours
15	53.2	4 hours
16	54.9	4 hours
17	52.2	4 hours
18	25.1	4 hours
19	79.4	4 hours
20	35.7	6 hours

For the Experiment 2, TMS-EEG and resting EEG recordings were performed at baseline (pre-drug) and at 2, 4 and 6 hours after drug intake (XEN1101). Blood samples were taken for every subject to evaluate drug plasma concentration. XEN1101 showed a pharmacokinetic profile characterized by a prolonged absorption and XEN1101 was detectable (<8.22 ng/mL) a week after administration, during the placebo experiment in those subjects who had placebo at the second visit. Therefore, to investigate XEN1101 effects, for each participant we selected post-dose measures for TMS-EEG and resting EEG measurements taken only during highest drug exposure (>8.22 ng/mL). Finally, we have performed a time matched placebo comparison at individual level. This means that if participant number 2 reached peak XEN1101 levels at 4hrs this was compared with the time-matched placebo condition at 4hrs.
